# Large Hysteresis effect in Synchronization of Nanocontact Vortex Oscillators by Microwave Fields

**DOI:** 10.1038/srep31630

**Published:** 2016-08-19

**Authors:** S. Perna, L. Lopez-Diaz, M. d’Aquino, C. Serpico

**Affiliations:** 1 University of Naples Federico II, Department of Electrical Engineering and Information Technology, I-80125 Naples, Italy; 2 University of Salamanca, Department of Applied Physics, Salamanca, Spain; 3 University of Naples “Parthenope”, Department of Engineering, I-80143 Naples, Italy

## Abstract

Current-induced vortex oscillations in an extended thin-film with point-contact geometry are considered. The synchronization of these oscillations with a microwave external magnetic field is investigated by a reduced order model that takes into account the dynamical effects associated with the significant deformation of the vortex structure produced by the current, which cannot be taken care of by using the standard rigid vortex theory. The complete phase diagram of the vortex oscillation dynamics is derived and it is shown that strong hysteretic behavior occurs in the synchronization with the external field. The complex nonlinear nature of the synchronization manifests itself also through the appearance of asymmetry in the locking frequency bands for moderate microwave field amplitudes. Predictions from the reduced order model are confirmed by full micromagnetic simulations.

Spin-transfer nano-oscillators (STNOs) based on nanocontact geometry are future candidates to develop nanoscale devices in the area of information and comunication technologies. Their current-field frequency tunability, narrow linewidth and stability at room temperature^1^,^2^ suggest their potential use in the microwave technology for realizing generators, detectors, modulators, etc. Furthermore, the micron size dimensions allow easy integration with the semiconductor technology. The main issue that limits their applicability is the low output power of the single device compared with LC voltage controlled oscillators^3^ (VCO). A potential solution to this issue is the synchronization of an arbitrary number of non interacting STNOs with a small amplitude external source, often referred to as injection-locking^4^. The synchronization of magnetization oscillations in nanopillars of submicron dimensions with nearly-uniform magnetization has been extensively studied in the last decade^4^,^5^,^6^,^7^,^8^,^9^,^10^, showing that the transitions among the possible regimes are governed by strongly nonlinear effects^11^,^12^,^13^,^14^,^15^. In particular, the hysteretic behavior of the synchronization mechanism, first predicted theoretically^16^,^17^,^18^ and later on observed experimentally^19^,^20^, allowed a step forward towards understanding the synchronization of STNOs by means of a ‘weak’ microwave source.

In this work, we study the synchronization mechanism between the dc current-induced vortex oscillations and a microwave external magnetic field in a thin film point-contact STNO^21^. These oscillation regimes in micron-sized thin-film STNO have been experimentally observed^22^,^23^ and theoretically studied mostly by means the rigid vortex theory^24^. The latter model describes a linear dependence of the vortex oscillation frequency on the current as well as a current independent distance of the vortex core from the center of the nanocontact. Neverthless, further experiments on vortex core polarity switching^25^,^26^ and vortex oscillations driven by magnetic field and spin polarized current^27^ were not in agreement with the predictions from the rigid vortex model. Indeed, numerical micromagnetic studies consistent with experimental results confirmed the presence of a vortex characterized by a deformed structure^28^,^29^. This effect, which is more pronounced close to the vortex core, has been also observed experimentally^30^.

Here we present a theoretical approach capable of taking into account the vortex structure deformation and apply the model to study the synchronization of current-driven magnetic vortex oscillations with an external microwave magnetic field for a disk-shaped point-contact STNO, as sketched in Fig. 1.

The main result of the work is the analytical derivation of the phase locking diagram (reported in Fig. 2(a,b)). This diagram describes all possible mechanisms of synchronization between vortex oscillations and external microwave magnetic field. Several insights emerge from our study. First of all, coexistence of synchronized and unsynchronized vortex oscillations is predicted for appropriate choices of amplitude and frequency of the microwave field. As a consequence of that, we show that the synchronization has a strong hysteretic nature. In addition, for microwave fields with moderately high amplitude (some tenths of mT), the locking frequency bands exhibit a pronounced asymmetry, which reveals complex nonlinear behaviour in the synchronization regime. The theoretical predictions are in good agreement with micromagnetic simulations of injection-locking experiments.

In the proposed theoretical model, vortex dynamics is modelled in terms of the vortex core position using a combined analytical-numerical approach. The analytical part consists of a collective variables description^31^ of the vortex core dynamics. The numerical one allows us to introduce an additional current dependence describing the vortex deformation that a current independent ansatz^24^,^32^ cannot take into account.

## Results

**Figure 1:**
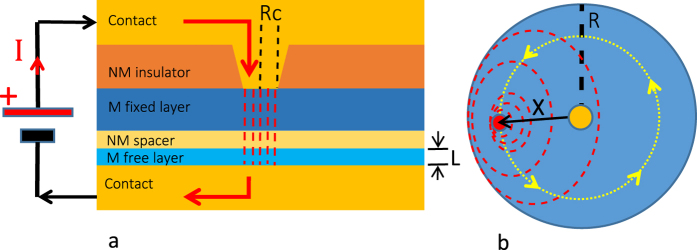
(**a**) Sketch of the point-contact STNO. (**b**) Sketch of the free layer. The orange dot located in the disk center is the cross-section of the nanocontact through which the electric current flows. The trajectory of the vortex core (red dot) around the nanocontact is represented by the yellow line. The dashed red lines represent the curling magnetization around the vortex core.

The nanocontact device under study is schematically represented in Fig. 1. The magnetic free layer has radius *R* = 1 *μm*, thickness *L* = 4 nm, and the radius of the nanocontact is *R*_*c*_ = 50 nm. Vortex oscillations are excited by injecting a current *I* through the nanocontact, which flows perpendicular to the free layer. We assume that the current density is confined in the free layer under the nanocontact region (see red dotted lines in Fig. 1). The fixed layer is assumed to be uniformly magnetized and tilted with respect to the out-of-plane direction by an angle *θ*_*p*_. This layer acts as spin polarizer for the current flowing through the multilayer structure. The microwave magnetic field is spatially uniform with amplitude *H*_*rf*_ and circularly-polarized in the free layer plane (*x*, *y*) with angular frequency *ω*.

**Figure 2:**
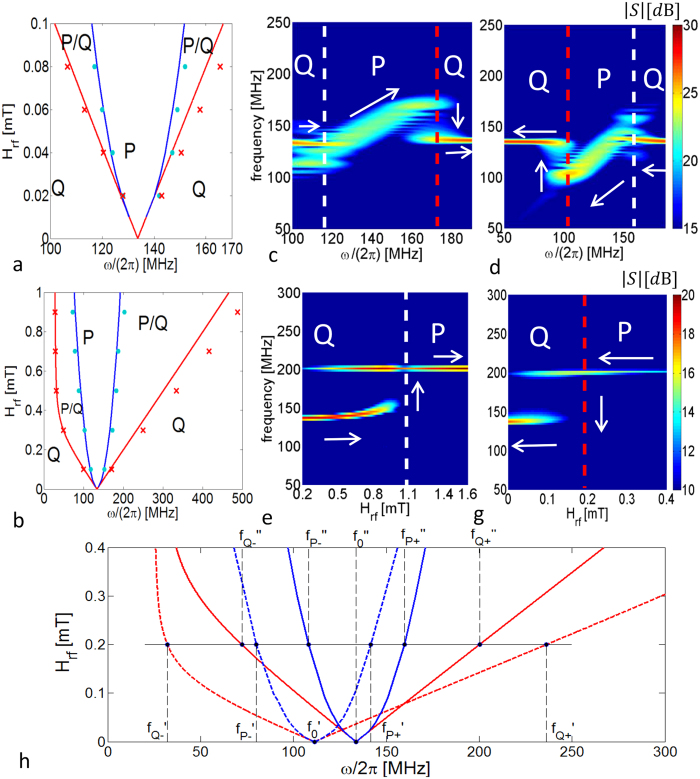
(**a**) phase locking diagram in the control plane (*ω*, *H*_*rf*_) for a spin-polarized current *I* = 10 mA with *θ*_*p*_ = 84°. Regions *P* and *Q* represent synchronized and unsynchronized states, respectively, whereas region *P*/*Q* indicates coexistence of both of them. Solid lines refer to reduced order model predictions, symbols to micromagnetic simulations; (**b**) extended view of the phase locking diagram for *μ*_0_*H*_*rf*_ < 0.1 mT; (**c**,**d**) spectrograms of the in-plane magnetization oscillation obtained from micromagnetic simulations of vortex dynamics at microwave field amplitude *μ*_0_*H*_*rf*_ = 0.1 mT and ramping the angular frequency *ω* up (**c**) and down (**d**); (**e**,**g**) spectrograms obtained at *ω*/(2*π*) = 200 MHz and ramping the field amplitude *H*_*rf*_ up (**e**) and down (**g**). The color scale refers to the spectrum amplitude expressed in *dB*; (**h**) superposition of the phase locking diagrams for different current values *I* = 5 mA (dotted lines) and *I* = 10 mA (solid lines).

In Fig. 2(a,b), we report the comparison of reduced order model and full micromagnetic simulations results for the phase locking diagram between the current induced vortex oscillations and the external microwave magnetic field.

The diagram represents all the possible stable oscillation regimes of the magnetic vortex as function of the control variables (*ω*, *H*_*rf*_). This diagram can be separated in three regions. Points in the region labelled with *P* correspond to periodic motions, termed *P*-modes, and are associated with vortex oscillations synchronized with the microwave magnetic field. On the other hand, points in the region labelled with *Q* correspond to quasi-periodic motions^33^, termed *Q*-modes, associated with unsynchronized vortex oscillations. In the region *P*/*Q* both synchronized (*P*-modes) and unsynchronized (*Q*-modes) regimes can exist for the same pair of control variables (*ω*, *H*_*rf*_). Which one of the two is reached in a particular situation depends on the past history of the vortex dynamical state. In this respect, the coexistence of multiple stable oscillation regimes implies the occurrence of hysteretic synchronization mechanisms.

The critical lines separating the different regions form two curved cones with a common vertex. The larger one, bounded by the red curves, separates the region where stable *P*-modes exist from the one where there are only unsynchronized *Q*-modes. The inner one, bounded by the blue curves, encloses the region where the only stable regimes are *P*-modes. The common vertex on the line *H*_*rf*_ = 0 mT corresponds to the free-running frequency *ω*_0_ of the nanocontact oscillator, namely the frequency of oscillations in the absence of microwave field.

To clarify the diagram, let us consider an experimental situation in which a microwave field of a certain amplitude has, initially, a very large frequency. According to the phase diagram in Fig. 2(a), the vortex oscillation regime in this situation will be identified with a point in the right region *Q* and, therefore, is unsynchronized. Then, let us suppose to slowly decrease the frequency while keeping the field amplitude constant, which means moving leftwards along a horizontal line. The system will stay in an unsynchronized regime (*Q*-mode) until the blue curve separating regions *P*/*Q* and *P* is crossed. At that point, the vortex oscillation will undergo synchronization with the microwave field, since the point (*ω*, *H*_*rf*_) enters the region *P* where the only stable regime is a *P*-mode. By further decreasing the frequency, the vortex oscillations will stay synchronized until the the red line separating the left *Q* and *P*/*Q* regions is crossed. When this occurs, the magnetic vortex will lose the synchronization with the microwave field and will move to a stable quasi-periodic motion. If the microwave field frequency is increased back, we move towards the right in Fig. 2(a) and the synchronization will be restored when the left blue line is crossed. By further increasing the frequency, the loss of synchronization will happen when the right red line is crossed. The hysteretic nature of the synchronization can be observed in the spectrograms presented in Fig. 2(c,d). They have been obtained from micromagnetic simulations by increasing (Fig. 2(c)) and decreasing (Fig. 2(d)) the frequency at constant rate of 100 KHz/ns. The amplitude of the rf field is 0.1 mT and the injected electric current is 10 mA. The red and white dashed lines indicate the *P* → *Q* and the *Q* → *P* transitions, respectively. It has been checked that smaller value of the frequency change rate does not affect the results.

We observe that the phase locking band, namely the width of region *P*, increases as a function of *H*_*rf*_ (see Fig. 2(a,b)). Moreover, for moderately high microwave field amplitudes, the frequency bands associated with coexistence of *P*/*Q* modes also increase and exhibit significant asymmetry, which is the signature of complex nonlinear dynamics. This featue has been already observed experimentally (for a wide range of frequencies) in point contact devices where the magnetization is nearly uniform^19^. Remarkably, we point out that such frequency bands are quite large (several tens of MHz for mT, above 100 MHz for *μ*_0_*H*_*rf*_ > 0.5 mT). Thus, it is expected that the hysteretic synchronization might be clearly observable even at room temperature, contrary to what happens for nanopillar STNOs^12^,^19^.

The hysteretic nature of the synchronization mechanism is also present if the frequency is maintained constant but the amplitude of the rf field is swept at a constant rate, which means moving vertically in the phase diagram of Fig. 2(a). It is observed in Fig. 2(e,f) where we present spectograms of the in-plane magnetization component obtained from micromagnetic simulations at 200 MHz sweeping the field up (e) and down (f) at a rate of 1 mT/ns in both cases. As in the previous case, it has been checked that a smaller rate does not affect the results.

In general, for generic histories of the microwave excitation, represented by closed curves in the control plane (*ω*, *H*_*rf*_), crossing the aforementioned critical lines might lead to hysteresis in the synchronization process. As outlined above, the different regions in the control plane (*ω*, *H*_*rf*_) are separated by curves associated with specific transitions of the vortex oscillation regimes. In particular, the blue curves describe transitions from *Q* → *P* (unsynchronized to synchronized regimes), whereas the red curves describe transitions *P* → *Q* (synchronized to unsynchronized regimes).

These critical curves, which can be analytically derived from our model, have been checked by means of full micromagnetic simulations. The results are visible in Fig. 2(a,b), where blue circle (red cross) symbols indicate the *Q* → *P* (*P* → *Q*) transitions observed in the simulations. The good quantitative agreement demonstrates the predictive power of the proposed theoretical model.

The wide range of frequencies around the free-running oscillation frequency in which synchronization is realized is an important requirement for the synchronization of multiple nanocontact vortex oscillators which, as it was pointed out in the introduction, is the solution to increase the power emitted by STNO devices. The study of synchronization of a family of nanocontact vortex oscillators goes beyond the limits of this work, but it is important here to carry out a general discussion about the simultaneous synchronization of multiple oscillators. The main point we want to stress is that, not only the region *P*, but also the region *P*/*Q* where synchronized and unsynchronized regimes coexist, is part of the frequency range in which simultaneous synchronization is possible.

In Fig. 2(h) we present the synchronization cones of two STNOs which differ in the free-running frequency (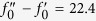 MHz) and we want to study the frequency range in which simultaneous synchronization occurs. The critical lines of the two STNOs have been computed considering the same STNO subjected to two different injected DC currents (*I* = 5 mA, *I* = 10 mA). This is done in order to simulate the situation where point-contacts are connected in parallel and there are differences in the contact resistances due to fabrication tolerances. The intersection points between the horizontal line corresponding to *H*_*rf*_ = 0.2 mT and the critical lines associated with the two oscillators are denoted with prime and double prime frequencies, respectively. Let us now consider again the experiment in which, starting from large enough frequency, we decrease it, i.e. we move from the right to the left on the horizontal line. The oscillator with the larger free-running frequency 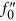 gets synchronized with the external field at the frequency 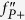, while the other one gets synchronized at the lower frequency 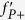. From frequencies below 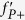, the oscillators are simultaneously synchronized down to the frequency 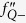 since 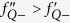). In this situation, the simultaneous locking frequency range is 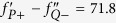 MHz, more than three times larger than the difference of the free-running frequencies 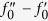. Remarkably, we observe that, in the absence of hysteresis, the locking range would be simply 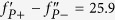 MHz, which is less than half of that achievable taking into account hysteresis.

Analogously, if we carry out the experiment by increasing the frequency at given field, by using the same line of reasoning, we find that the frequency range of simultaneous synchronization between the two STNOs goes from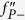 to 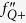 which, according to our computations, is equal to 92.1 MHz. We notice that, in this case, the frequency 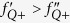 and, thus, the second one determines the upper bound.

In conclusion, the hysteretic synchronization, coupled with an appropriate scheme of variation of the excitation conditions, may lead to considerably large frequency ranges where simultaneous synchronization of nanocontact STNOs is possible.

### Theoretical modelling of microwave field driven phase-locked vortex oscillation

The magnetization dynamics in the free layer of the point-contact structure depicted in Fig. 1 is described by the Landau-Lifshitz-Gilbert equation^34^ properly generalized with the Slonczewski spin transfer torque^35^, referred in the following as LLGS equation:


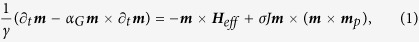


where ***m*** is the magnetization unit-vector with ***m*** = ***M***/*M*_*s*_, *γ* is the absolute value of the gyromagnetic ratio, *α*_*G*_ is the Gilbert damping constant, *J* is the current density, *σ* is the spin transfer torque efficiency. The effective field, expressed as variational derivative, is ***H***_*eff*_ = −*δG*_*L*_/*δ*(*μ*_0_***m**M*_*s*_*V*) where *V* is the free layer volume and


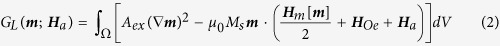


is the micromagnetic free energy expressed as sum of the exchange, magnetostatic and Zeeman contributions, respectively. We consider the case where the applied field ***H***_*a*_ is zero, which means that the only contribution to the Zeeman energy is due to the Oersted field generated by the current. Assuming that the free layer is in a vortex state and starting from the LLGS eq. (1), a reduced order model where the vortex dynamics is described in terms of the vortex core center coordinates ***X*** = (*X*_1_, *X*_2_) can be derived by using a collective variables description^31^ such that ***m***(***r***, *t*) = ***m***(***r***, ***X***(*t*)). With this approach, one arrives at the following equation:


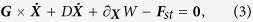


where ***G*** = *G**e***_*z*_ is the gyrovector directed along z, *D* is the damping coefficient, *W* = *W*_*ex*_ + *W*_*m*_ + *W*_*Oe*_ is the total energy of the vortex in terms of exchange, magnetostatic and Zeeman-Oersted energies, and ***F***_*st*_ is the spin torque force term. Eq. (3) can be also viewed as a Thiele-like model^36^ for the vortex dynamics in the thin-disk layer. Due to the rotational symmetry of the system, all these quantities depend just on the amplitude of ***X***. In the case of conservative dynamics (*α*_*G*_ = 0, *D* = 0, *F*_*st*_ = 0), the equation describing the vortex motion is:


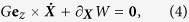


which describes a circular motion around the nanocontact with orbit radius *X* and precession frequency *f*(*X*, *I*) given by:


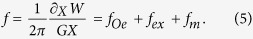


**Figure 3:**
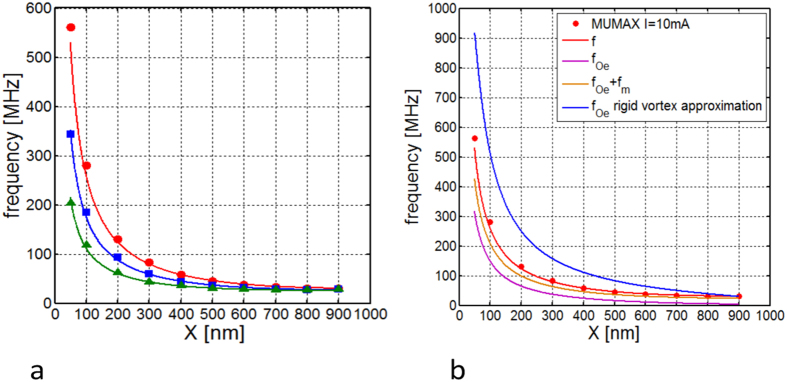
Comparison between the vortex oscillation frequency estimated with full micromagnetic simulations and evaluated with reduced order model for different current values. (**a**) The red circles, blue squares and green triangles are obtained from MUMAX^38^ for *I* = 10 mA, *I* = 5 mA and *I* = 2.5 mA respectively. The continuous line of the same color is related to the same current value but is obtained from eq. (5) using the polynomial expression for *W*(*X*, *I*). (**b**) Different contributions to the total frequency *f* according to eq. (5). The blue curve represents the frequency estimated according to eq. (5) under the assumption of rigid vortex profile for the magnetization distribution and considering only the Zeeman energy due to the Oersted field.

The gyrovector module is *G* ≈ 2*πpμ*_0_*M*_*s*_*L*/*γ*, where *p* is the vortex core polarity, while the total energy is approximated with the following fourth degree polynomial *W*(*X*, *I*) = *W*_0_ + *c*_1_*X* + *c*_2_*X*^2^ + *c*_4_*X*^4^, where the coefficients are obtained with a least squares fitting to the results of full micromagnetic simulations of the conservative dynamics (see Methods). In Fig. 3(a), we show that the reduced order model where the current dependence of the energy *W*(*X*, *I*) is identified from micromagnetic computation of the energies (see section Methods) is in good agreement with the oscillation frequency resulting from full micromagnetic simulations of conservative vortex gyration. Furthermore, in Fig. 3(b) one can see that each contribution (exchange *f*_*ex*_, Oersted *f*_*Oe*_ and magnetostatic *f*_*m*_) is important for the correct estimation of the conservative gyration frequency. In addition, it is important to notice that the rigid vortex theory is not able to reproduce the vortex dynamics in this case. In fact by looking at the blue line in Fig. 3(b) computed assuming a rigid vortex state with *W*(*X*, *I*) ≈ *W*_*Oe*_(*X*, *I*) ⇔ *f*(*X*, *I*) ≈ *f*_*Oe*_(*X*, *I*)^22^,^24^, it is apparent that the frequency so predicted overestimates the frequency estimated by full micromagnetic simulations by a large amount.

**Figure 4:**
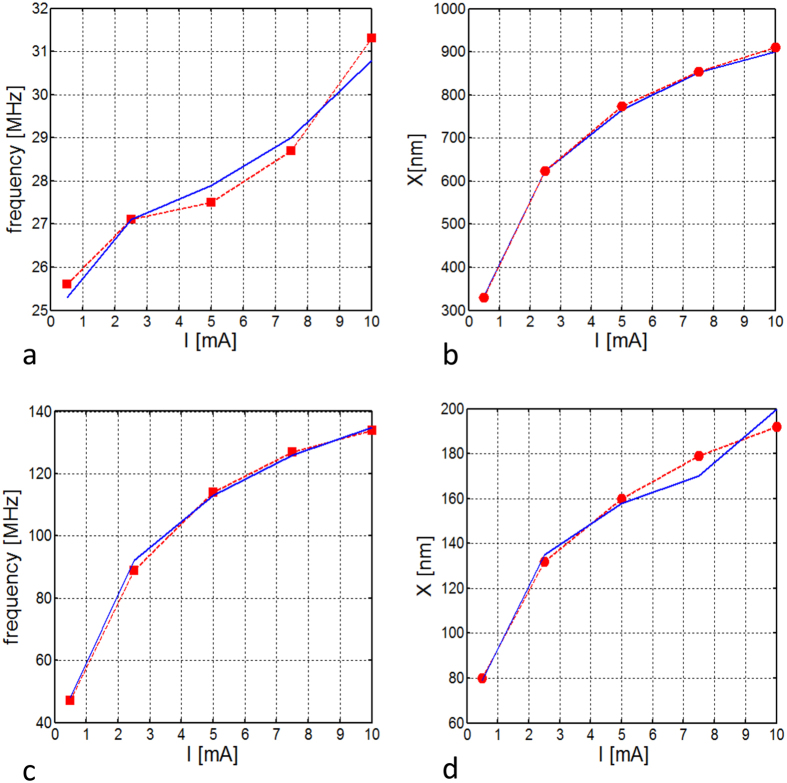
Oscillation frequency *f* and stable orbit radius *X* as function of the spin-polarized current. (**a**) *f*(*I*), (**b**) *X*(*I*) for *θ*_*p*_ = 0 (out-of-plane polarizer); (**c**) *f*(*I*), (**d**) *X*(*I*) for *θ*_*p*_ = 84° (in-plane polarizer). Solid lines are obtained from eq. 8, whereas lines with symbols refer to the results of full micromagnetic simulations.

When damping and spin torque effects are introduced, the vortex dynamics can be described by eq. (3) in polar coordinates (*X*, Φ):


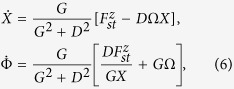


where


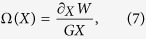


is again the angular frequency of the vortex core oscillating at constant *X*. In fact, considering the case of stationary oscillation 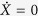, one has


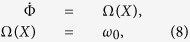


where 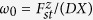. In the rigid vortex theory^24^, the term *D* does not depend on the current, while both Ω and *ω*_0_ depend linearly on it. Consequently, the stationary orbit radius *X* of the vortex core precession around the nanocontact is current-independent. From micromagnetic simulation of vortex oscillation driven by spin polarized current shown in Fig. 4 we observe a total disagreement with the rigid vortex theory predictions. In particular, in both cases with out-of-plane (see Fig. 4(a,b)) and almost in-plane (see Fig. 4(c,d)) polarizer, the dependence *X*(*I*) is not negligible and the oscillation frequency is not linear with respect to the current value.

**Figure 5:**
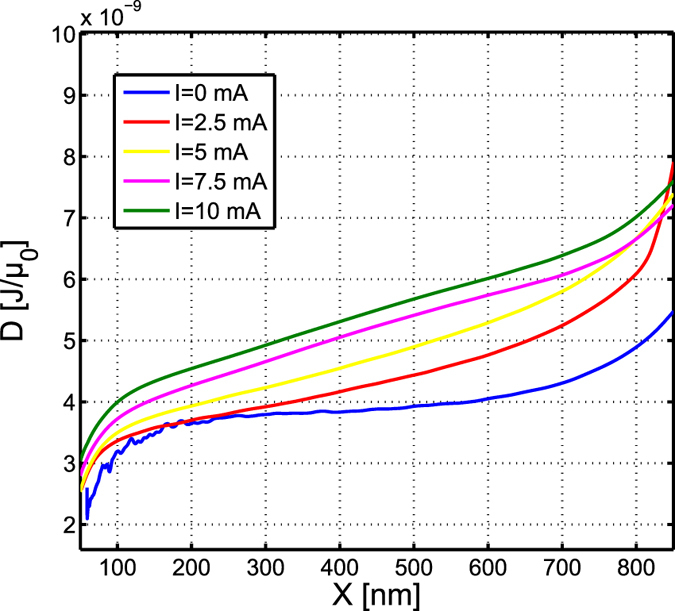
Damping *D* as a function of *X* for several values of the current *I*.

In order to reproduce such features, we found out that it is necessary to fit both the energy *W* and the damping term coefficient *D* with micromagnetic simulations. The identification procedure of *D* can be inferred from the reduced order model described by eq. (3) in the absence of spin torque force ***F***_*st*_ = **0**. In this framework, we end up with the following equation:


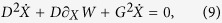


which is a second degree polynomial equation in *D* that can be solved once 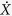 is known. This last term is obtained from a numerical experiment of relaxation dynamics. In particular, we simulate the vortex core dynamics starting from the initial condition where the vortex core is displaced from the center and we compute its dynamics towards equilibrium (center of the nanocontact) monitoring the energy *W*(*t*, *I*). From the knowledge of *W*(*X*, *I*), *X*(*t*) is extracted. Since *D*^2^/*G*^2^ ≪ 1, we can approximately estimate 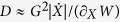. At this point, the knowledge of *W*(*X*, *I*) and *X*(*t*) allows us to obtain *D*(*X*, *I*) from the solution of eq. (9). In this way, we get two roots, but a simple comparison of their order of magnitude with the one of the approximate value allows one to select the correct one. We remark that the characterization of the damping term coefficient does not involve the spin torque force term. In Fig. 5, the behavior of *D*(*X*, *I*) obtained following the procedure described above, is reported. We observe that, while for *I* = 0 mA *D*(*X*) is approximately constant, the dependence on *X* becomes more important as the current increases.

For the spin torque force term, one can infer that, for an arbitrary orientation of the polarizer, only the out-of-plane component can compensate the effect of damping. We adopt for this term the same expression as that provided by the rigid vortex theory^24^, namely 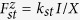, where *k*_*st*_ = *MsσIL*cos*θ*_*p*_.

After the identification of *W*(*X*, *I*) and *D*(*X*, *I*), from the resolution of eq. (8), we get the blue curves in Fig. 4. As can be oserved, they are in very good agreement with full micromagnetic simulations (red symbols).

Up to this point, our model describes a scenario where the only external source exciting the vortex dynamics is the dc spin polarized current flowing through the nanocontact. In what follows, we generalize our model in order to include the presence of a microwave magnetic field, instrumental for the synchronization study. We model the interaction between the microwave field and the vortex magnetization state with an additional Zeeman energy contribution. This energy term can be evaluated as:


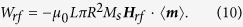


where 〈⋅〉 means spatial average and


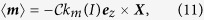


where 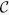 is the chirality of the vortex and the proportionality constant *k*_*m*_ depends on the current *I* and is evaluated from numerical simulations. This interaction appears in the model through its gradient with respect the vortex core position vector ***X***:


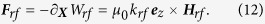


where 
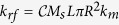
.

Thus, the equations which describe the vortex motion become:


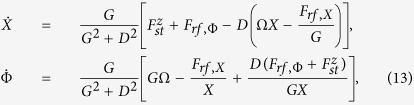


where


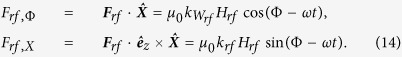


Here, we use the approach proposed in refs 11,17 where the dynamical equations are rewritten in an appropriate rotating frame leading to an autonomous (i.e. time-independent) two-dimensional system. As a consequence of that, we can completely characterize the dynamical state by means of bifurcation theory^17^,^37^. Let us consider a rotating reference frame with angular frequency *ω*. In this frame, the angular polar coordinate Φ = *ϕ* + *ωt* and the rotating field components are given by:


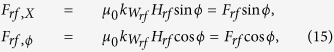


which, as mentioned before, are time-independent.

Then, if we introduce the normalized quantities *d* = *D*/*G*, *x* = *X*/*R*, *b* = *F*_*rf*_/(*GR*), 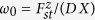 and neglect the 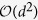 terms, eq. (13) projected in the rotating frame take the following form:


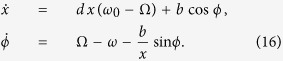


The latter equations reveal a simple, but neverthless rich mathematical structure, as we will see in the following. In fact, from general arguments of nonlinear dynamical system theory^37^, it is possible to state that the only steady-state solutions admitted by eq. (16) are either stationary points or limit cycles. As far as the synchronization with the rotating field is concerned, we pay special attention to the equilibrium states. In fact, such equilibria in the rotating reference frame represent synchronized motions of the vortex with the rotating field (*P*-modes) if we interpret them in the lab reference frame. Conversely, limit cycles in the rotating frame represent unsynchronized quasi-periodic vortex motions (*Q*-modes) in the lab frame.

**Figure 6:**
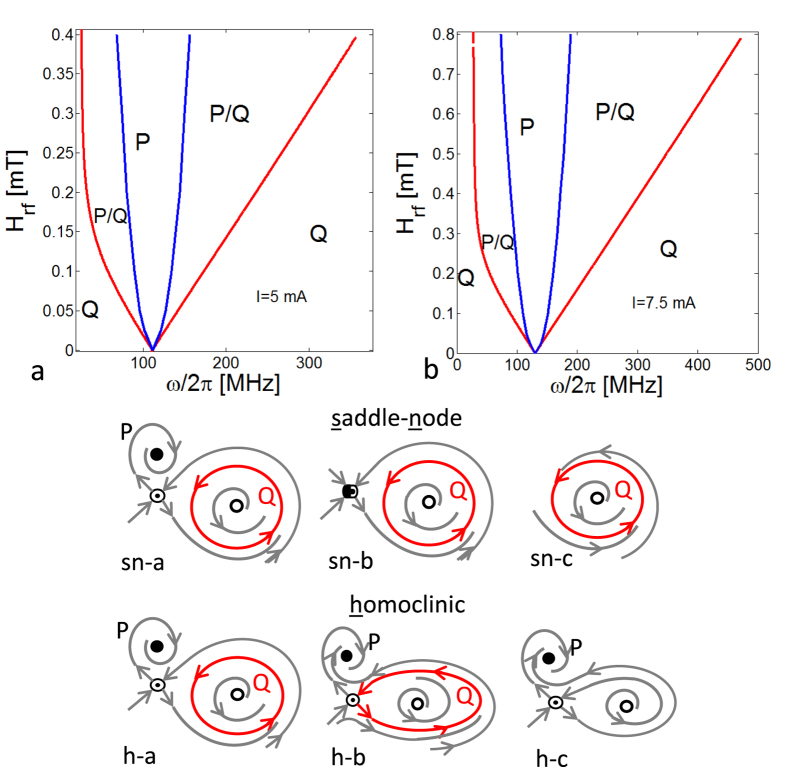
Phase locking diagrams in the control plane (*ω*, *H*_*rf*_) as function of current. (**a**) *I* = 5 mA; (**b**) *I* = 7.5 mA; red lines refer to saddle-node bifucations and blue lines refer to homoclinic bifurcations; (sn) qualitative sketches for saddle-node bifurcations, the sequence (**a**–**c**) reproduces the generic transition from region *P*/*Q* to region *Q*; (**h**) qualitative sketches for homoclinic bifurcations, the sequence (**a**–**c**) reproduces the generic transition from region *P*/*Q* to region *P*.

All the possible transitions between P ↔ Q modes are a consequence of a bifurcation process^37^. Among the possible bifurcations for a two-dimensional dynamical system, for the amplitude of current (2.5 mA ≤ *I* ≤ 10 mA) and microwave field (*H*_*rf*_ ≤ 1 mT) considered, we find out that, for the dynamical system described by eq. (16), just two of them occur as function of the current: the saddle-node and the homoclinic bifurcation. However this does not exclude the presence of other bifurcations that might occur at higher microwave power. A sketch of these two bifurcation mechanisms is reported in Fig. 6(sn,h), where generic transitions from region *P*/*Q* to regions *Q* and *P* are described. In the former case, an unstable *P*-mode (saddle point, represented as a striped dot in figure) annihilates with a stable synchronized state (filled dot) leaving just a stable *Q*-mode (red closed curve) as admissible regime. In the case of homoclinic bifurcation, a stable unsynchronized state (*Q*-mode) disappears through a saddle connection, leaving just a stable synchronized *P*-mode as admissible regime. From the description of these bifurcation mechanisms, one can conclude that in the control plane the saddle node bifurcations are responsible for the P → Q transitions, whereas homoclinic bifurcations are responsible for the Q → P ones.

Now we address the quantitative determination of the bifurcation curves from eq. (16). The first step is to express the synchronization conditions:


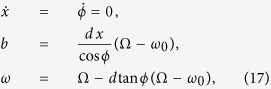


where a correspondence between the synchronized states in the rotating frame (*x*, *ϕ*) and the points of the control plane (*ω*, *H*_*rf*_) is established. The saddle node bifurcation curve can be obtained by considering the linearized form of eq. (16) 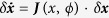, being *δ**x*** = (*δx*, *δϕ*), ***J***(*x*, *ϕ*) the Jacobian matrix of the right hand side of (16), evaluated in the equilibrium point (*x*, *ϕ*). In particular, by solving eq. (17) together with det{***J***(*x*, *ϕ*)} = 0, we obtain the red curves in Fig. 6(a,b). On the other hand, as far as the homoclinic bifurcation is concerned, there are no general approaches since it is a global bifurcation. Here we detect the occurrence of such a bifurcation from the analysis of the phase portrait of the dynamical system (16) in the plane (*x*_1_ = *x*cos*ϕ*, *x*_2_ = *x*sin*ϕ*). By keeping *ω* fixed while changing *b*, we determine the critical value of this parameter related with the onset of the homoclinic bifurcation, as sketched in Fig. 6(h-a,h-b,h-c), by following a bisection method. By repeating this procedure, we obtain the blue curves enclosing the region *P* in the phase locking diagram of Fig. 6(a,b).

The structure of the phase locking diagram is preserved when the current value *I* changes, as shown in Fig. 6(a,b). This confirms the general character of the overall picture of the vortex dynamics outlined above in the considered current range.

## Discussion

The quantitative predictive power of the proposed model is based on appropriate generalization of the Thiele-like model relying on the rigid vortex ansatz^22^,^24^. In fact, the central and distinctive feature of our modelling is the introduction of the current dependence in the energy and damping terms *W*(*X*, *I*), *D*(*X*, *I*) within the framework of the collective variables description (see eq. (3)).

**Figure 7:**
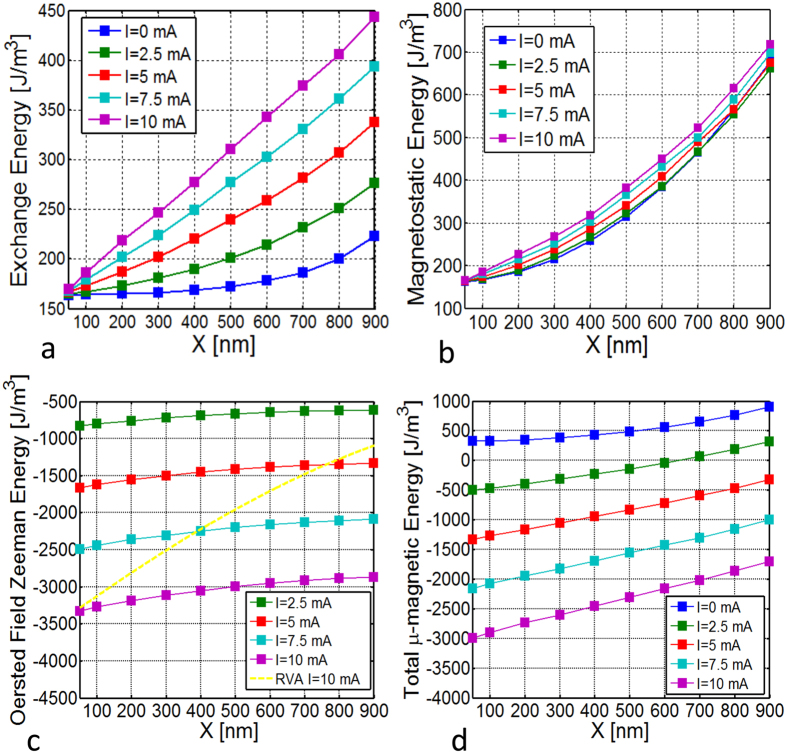
Energies as function of *X* and *I* computed from micromagnetic simulations. (**a**) Exchange energy; (**b**) magnetostatic energy; (**c**) Zeeman energy; (**d**) total energy. Solid lines of different colors refer to different values of *I*, dashed yellow line in (**c**) refers to the Zeeman energy predicted by a rigid vortex description.

In fact, such a dependence is necessary in order to reproduce the actual behavior of the stationary vortex core orbit radius *X* under the action of the spin-polarized current *I*, as well as the free running oscillation frequency *f*(*I*) of the STNO, as shown in Fig. 4, where the comparison of theory and micromagnetic simulations is reported for two different orientations of the polarizer: out-of-plane (*θ*_*p*_ = 0°) and in-plane (*θ*_*p*_ = 84°). We point out that an out-of-plane component of the spin-torque force is always required in order to compensate the damping. Micromagnetic analysis shows that the rigid vortex theory cannot be applied for a quantitative description of nanocontact STNOs. In Fig. 7, the behavior of the different energy terms as function of *X* and *I* is reported. One can clearly see that the exchange and the magnetostatic energy (Fig. 7(a,b)) which, within the context of a rigid vortex model are considered to be independent of the vortex core position and current, exhibit monotonically increasing behavior as function of *X*, but also with respect to *I* in a nonnegligible way. In addition, the Zeeman energy associated with the Oersted field (Fig. 7(c)) shows a weak dependence on *X* compared to what a rigid vortex description would predict (see yellow curve).

**Figure 8:**
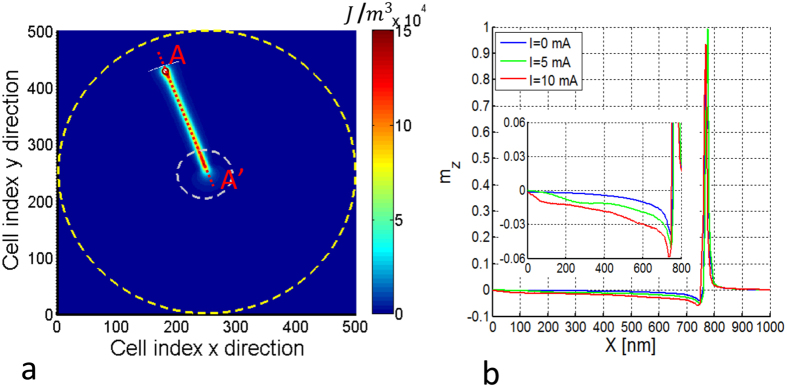
(**a**) Exchange energy density of the vortex configuration for *I* = 10 mA and *X* = 800 nm; (**b**) *m*_*z*_ along the line A-A’ represented in (**a**) for different current values *I*. Inset: magnified view.

The strong dependence on the current can be also explained by looking at the micromagnetically computed magnetization configuration, where a strong deformation of the vortex magnetization pattern is clearly visible (see Fig. 8(b)). The in-plane deformation is responsible for the weak dependence on *X* of the Zeeman energy and for the dependencies on both *X* and *I* of the exchange energy (see Fig. 7(a,c)). In fact, while for large enough distance from the vortex core the magnetization aligns with the Oersted field, in a small region around the line connecting the core center and the nanocontact center (line A-A’ in Fig. 8(a)) there is a sharp change of the magnetization direction. This change of direction is associated with a consistent amount of exchange energy stored along this branch structure, as in Fig. 8(a) where the exchange energy density is represented.

We also observe that the deformation of the vortex structure affects not only the in-plane magnetization components *m*_*x*_, *m*_*y*_, but also *m*_*z*_. In fact, one can see in Fig. 8(b) that, as far as the current is increased, in the region along the line A-A’ except the vortex core region, the out-of-plane magnetization *m*_*z*_ exhibits a nonzero tilting downwards which increases with *X* and *I*. This deformation of the vortex structure can explain the current dependence of the magnetostatic energy reported in Fig. 7(b).

We can conclude that the deformation of the magnetic vortex is more pronounced as far as the current is increased and, therefore, it is essential to take it into account in a collective variables description.

## Methods

### Identification of vortex state energy from micromagnetic simulations

The micromagnetic analysis of the vortex dynamics is performed with MUMAX^38^. We simulate the vortex core precession around the point-contact, for given current value *I* non spin polarized and for Gilbert damping *α*_*G*_ = 1.3 × 10^−3^, starting at a distance *X* from the disk center. For such a low value of the damping, the vortex core dynamics can be approximately considered as occurring at constant energy for sufficiently long time in order to identify the functions *W*_*ex*_(*X*, *I*), *W*_*m*_(*X*, *I*), *W*_*Oe*_(*X*, *I*) and *W*(*X*, *I*) reported in Fig. 7.

### Micromagnetic simulations of injection locking

From a systematic micromagnetic analysis, it is possible to reproduce a numerical synchronization map. The generic simulation describes the excitation of a stable vortex oscillation around the nanocontact driven by a microwave rotating field. In the computations, the material parameters are: saturation magnetization *M*_*s*_ = 870 kA/m, exchange constant *A*_*ex*_ = 1.3 × 10^−11^ J/m, Gilbert damping *α*_*G*_ = 0.013 and polarization factor *P* = 0.5. The free layer is discretized into cells of 4 × 4 × 4 nm^3^ (the exchange length of the material is 5.23 nm). In the absence of microwave field, *H*_*rf*_ = 0, the vortex gyrates around the nanocontact with free-running frequency *f* = 135 MHz.

The rotating magnetic field has given amplitude *H*_*rf*_ and angular frequency *ω* that increases at constant rate. For each given value of *H*_*rf*_, we make two different simulations. The first one where 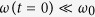 and 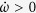. Conversely, in the second one we have 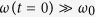 and 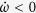. Starting from an unsynchronized condition, from these simulations we get the values of *ω* at which the vortex oscillations begin to synchronize/unsynchronize. In this way, for each field value, we obtain the four numerical points shown in Fig. 2(a,b). The synchronized state is detected from the spectrograms of the 〈*m*_*x*_〉 (*t*) (〈·〉 means spatial average) like those in Fig. 2. The single spectrogram is obtained by doing a short-time Fourier transform of 〈*m*_*x*_〉 (*t*).

## Additional Information

**How to cite this article**: Perna, S. *et al*. Large Hysteresis effect in Synchronization of Nanocontact Vortex Oscillators by Microwave Fields. *Sci. Rep*. **6**, 31630; doi: 10.1038/srep31630 (2016).
